# Bilateral Lung Artery Embolization Mimicking an Acute Myocardial Infarction

**DOI:** 10.1155/2021/6616139

**Published:** 2021-06-15

**Authors:** Maria Paparoupa, Razaz Aldemyati, Myrto Theodorakopoulou

**Affiliations:** ^1^Department of Intensive Care Medicine, University Medical Center Hamburg-Eppendorf, Martinistr.52, Hamburg 20246, Germany; ^2^Rabigh Faculty of Medicine, King Abdulaziz University, Jeddah 21589, Saudi Arabia

## Abstract

Electrocardiographic abnormalities in patients with massive pulmonary embolism are common and unspecific. An 80-year-old woman was admitted to our department with severe respiratory insufficiency and hemodynamic instability. Abnormal high-sensitivity cardiac troponin I and ST-segmental elevation in II, III, aVF, and V3–V6 were present on admission. Segmental motion abnormalities of the left ventricular wall were not detectable in echocardiography. Instead, the presence of a right ventricular strain raised the suspicion of a lung artery embolization. The diagnosis was confirmed by a computed tomography of the chest, and a thrombolytic therapy with 100 mg recombinant tissue plasminogen activator (rt-PA) was administered. Though respiratory and hemodynamic stability were established, electromechanical disassociation suddenly occurred 30 hours later and the patient died. Electrocardiographic changes mimicking a myocardial infarction may occur after a massive pulmonary embolism and constitute a diagnostic challenge for clinicians being active in the field of emergency medicine and intensive care.

## 1. Introduction

Since pulmonary arterial imaging emerged as the main diagnostic tool of acute pulmonary embolism, electrocardiographic findings have lost their importance in diagnosing this life-threatening condition [1]. Although newly introduced electrocardiographic trends have been proposed to consist valuable diagnostic tools in patients with pulmonary artery embolization [2, 3], well-established electrocardiographic changes are mainly implemented as prognostic indicators of clinical outcome, such as development of a secondary pulmonary hypertension, circulatory shock, and in-hospital mortality [4–6]. We present a case of a bilateral lung artery embolization mimicking an acute myocardial infarction.

## 2. Case Presentation

**Figure 1:**
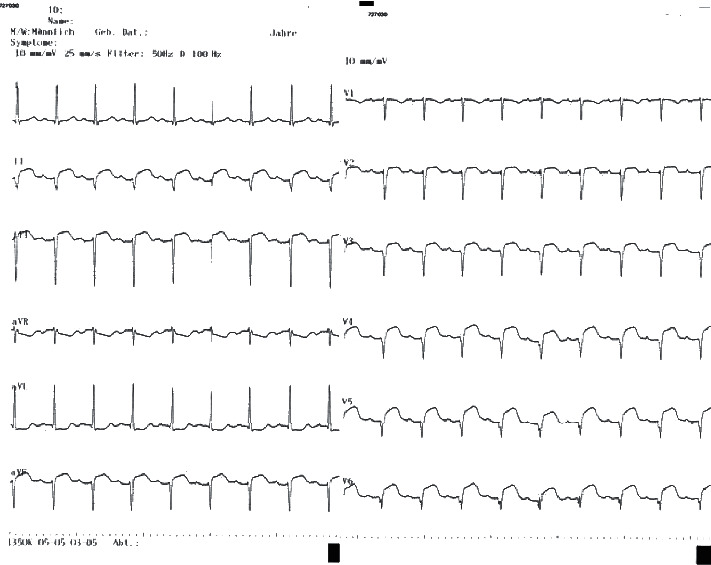
12-lead electrocardiogram (ECG) on admission showing an ST-segment elevation in II, III, aVF, and V3–V6.

**Figure 2:**
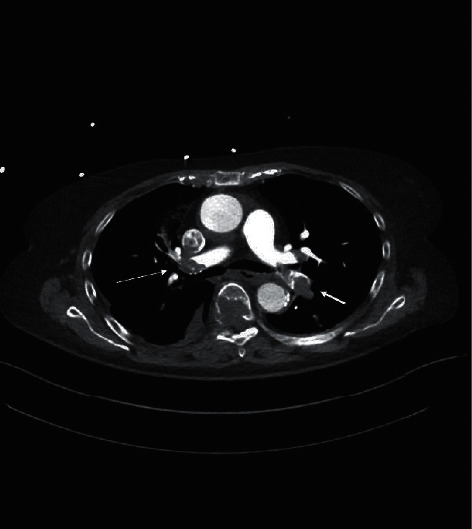
Computed tomography (CT) scan of the chest, showing the bilateral pulmonary artery embolization (PAE) (the thin arrow on the right side and the thick arrow on the left side).

A woman in her 80s, with no history of cardiovascular disease or cardiovascular risk factors, was admitted to our territory medical center with acute dyspnea and sinus tachycardia of 1 hour's duration. Further symptoms were not assessable, due to an underlying dementia. Shortly before admission, she had a syncope and systemic arterial hypotension of 80/50 mmHg. On her arrival to our emergency department, her heart rate war regular at an average of 110 bpm, respiration rate was 35 per minute, and peripheral oxygen saturation (SpO2) was 80% on ambient air. Results of blood chemistry tests and complete blood cell count revealed a serum lactate of 10 mmol/L (normal range 0.5–1.0 mmol/L) without further abnormalities. The initial high-sensitivity cardiac troponin I (hs-cTnI) was 1027 pg/ml (normal range <400 pg/ml), and ST-segment elevation was present in II, III, aVF and V3–V6 of a 12-lead electrocardiogram (ECG) (Figure 1). A bedside echocardiography revealed an acute right ventricular strain, but left ventricular systolic function was normal, without segmental wall motion abnormalities. A computed tomography (CT scan) of the chest confirmed the diagnosis of a bilateral pulmonary artery embolization (PAE) (Figure 2). A rescue thrombolytic therapy with 100 mg recombinant tissue plasminogen activator (rt-PA) was administered intravenously. 12 hours later, respiratory stability was achieved and the patient remained hemodynamically stable without catecholamine. Nevertheless, ST-segment elevation persisted and hs-cTnI was still above the normal range with 40.580 pg/ml. A repeated bedside echocardiography revealed improvement of the right ventricular strain and left ventricular systolic function remained intact. However, 30 hours later, electromechanical disassociation suddenly occurred, and the patient died.

## 3. Discussion

Severe respiratory insufficiency and hemodynamic instability occurred after bilateral pulmonary artery embolization in our case. Abnormal hs-cTnI and ST-segmental elevation in II, III, aVF, and V3–V6, otherwise signs of an acute myocardial infarction, were interpreted in the context of the concurrent right ventricular strain, which was present in echocardiography. Traditional electrocardiographic manifestations of acute cor pulmonale, such as SI QIII or SI QIII TIII pattern, right bundle branch block, P pulmonale, or rightward QRS complex axis shift were absent in the patient's ECG [7]. According to a retrospective study by Stein PD et al., the most common electrocardiographic abnormalities in patients with acute submassive or massive pulmonary embolism are nonspecific T-wave changes and nonspecific elevation or depression of the RST segment [8]. Ischemic signs, mimicking acute coronary syndrome, have been also described in the literature [9, 10].

Because segmental motion abnormalities of the left ventricular wall were absent in the admission echocardiography of our case, we avoided performing an emergency coronary angiography, even though both electrocardiographic changes and the elevation of hs-cTnI denoted an ongoing ischemic myocardial trauma. We supposed that the underlying state of severe systemic hypoxemia caused a global myocardial tissue hypoperfusion, which was rather functional than anatomical as observed in conditions of a coronary artery occlusion due to arterial thrombosis.

An additional argument for not performing an emergency percutaneous coronary intervention (PPCI) is that the administered thrombolytic therapy already consisted a validated treatment in the hypothetical case of an underlying acute myocardial infarction, and therefore, both conditions were concurrently treated with this therapeutical approach [11].

After thrombolytic therapy was administered, hs-cTnI remained detectable in abnormal levels and ST-segment elevation was present in serial electrocardiographic controls. The persistence of elevated heart enzymes and abnormal electrocardiographic findings up to two weeks after pulmonary artery embolization is a well-described phenomenon and, therefore, raised no worries in the medical team, regarding the issue of a prolonged myocardial hypoperfusion [2].

However, the coronary status of the patient and the presence of a concurrent myocardial infarction remain uncertain, as a postmortem examination has been opposed from the patient's relatives. Whether an early coronary angiography could have influenced the clinical course of our case is debatable. The authors believe that even if an acute myocardial infarction was present, thrombolytic therapy was still indicated as the primary treatment option because shock and respiratory insufficiency, due to the underlying pulmonary embolism, were the main clinical manifestations, presenting a vital threatening condition on admission.

## 4. Conclusions

Electrocardiographic changes mimicking a myocardial infarction may persist after a massive pulmonary embolism and constitute a diagnostic challenge for clinicians being active in the field of emergency medicine and intensive care.

## Data Availability

All data analyzed during this study are included in this manuscript.

## Abbreviations

SpO2: Peripheral oxygen saturation
hs-cTnI: High-sensitivity cardiac troponin I
ECG: Electrocardiogram
CT scan: Computed tomography scan
PAE: Pulmonary artery embolization
rt-PA: Recombinant tissue plasminogen activator
PPCI: Primary percutaneous coronary intervention.
